# Supplementary material for: Splinting in horizontal root fractures: A Bayesian network meta-analysis
Source: PLoS One. 2025 Jun 26;20(6):e0326979. doi: 10.1371/journal.pone.0326979 (PMC12200830; doi:10.1371/journal.pone.0326979)
Supplement: S1 Appendix — (DOCX) [file pone.0326979.s001.docx]

**Appendix 1 -** Database search strategy.

| **Database** | **Search** |
| --- | --- |
| **Lilacs** | ("tooth injuries" OR "Teeth Injury" OR "tooth injury" OR "teeth injuries" OR "Tooth Fractures" OR "Tooth Fracture" OR "Horizontal root fracture" OR "Transverse Root Fracture" OR "root fracture" OR "dental trauma" OR "dental injuries" OR "dental injury" OR "Dentoalveolar trauma" OR "intraalveolar root fracture" OR "intra alveolar root fracture" OR "lesões nos dentes" OR "trauma nos dentes" OR "Fraturas nos dentes" OR "Fratura no dente" OR "Fratura horizontal da raiz" OR "Fratura transversal da raiz" OR "fratura radicular" OR "traumatismo dentário" OR "lesões dentárias" OR "lesão dentária" OR "Trauma dentoalveolar" OR "fratura de raiz intraalveolar" OR "lesiones en los dientes" OR "fracturas de los dientes" OR "fractura de diente" OR "Fractura horizontal de la raíz" OR "Fractura transversal de la raíz" OR "fractura de la raíz" OR "trauma dental" OR "lesiones dentales" OR "lesión dental" OR "Traumatismo dentoalveolar" OR "fractura de raíz intraalveolar") AND ("splints" OR "splint" OR "Static Splints" OR "retention" OR "splinting" OR "Flexible splinting" OR "types of splints" OR "rigid splinting" OR "rigid splint" OR "flexible splint" OR "semi-rigid splinting" OR "tooth stabilization" OR "tooth splinting" OR "splinted" OR "esplintagem" OR "esplintagem estáticas" OR "retenção" OR "esplintagem flexíveis" OR "tipos de esplintagem" OR "esplintagem rígida" OR "esplintagem rígidas" OR "contenção dental" OR "contenção flexivel" OR "contenção semirrígida" OR "contenção rígida" OR "esplintagem flexível" OR "esplintagem semirrígida" OR "estabilização do dente" OR "esplintagem dentária" OR "Férulas" OR "férula" OR "Férulas estáticas" OR "retención" OR "Férula flexible" OR "tipos de férulas" OR "férula rígida" OR "férula flexible" OR "férula semirrígida" OR "estabilización de dientes" OR "férula de dientes" OR "entablillado") |
| **PubMed** | 1. (“tooth injuries”[MeSH Terms] OR “tooth injuries”[All Fields] OR “Teeth Injury”[All Fields] OR “tooth injury”[All Fields] OR “teeth injuries”[All Fields] OR “Tooth Fractures”[MeSH Terms] OR “Tooth Fractures”[All Fields] OR “Tooth Fracture”[All Fields] OR “Horizontal root fracture”[All Fields] OR “Transverse Root Fracture”[All Fields] OR “root fracture”[All Fields] OR “dental trauma”[All Fields] OR “dental injuries”[All Fields] OR “dental injury”[All Fields] OR “Dentoalveolar trauma”[All Fields] OR “intraalveolar root fracture”[All Fields OR “intra alveolar root fracture”[All Fields) 2. (“Splints”[MeSH Terms] OR “splints”[All Fields] OR “splint”[All Fields] OR “Static Splints”[All Fields] OR “retention”[All Fields] OR “splinting”[All Fields] OR “Flexible splinting”[All Fields] OR “types of splints”[All Fields] OR “rigid splinting”[All Fields] OR “rigid splint”[All Fields] OR “flexible splint”[All Fields] OR “semi-rigid splinting”[All Fields] OR “tooth stabilization”[All Fields] OR “tooth splinting”[All Fields] OR “splinted”[All Fields]) 3. #1 AND #2 |
| **SCOPUS** | TITLE-ABS-KEY ( "tooth injuries" OR "Teeth Injury" OR "tooth injury" OR "teeth injuries" OR "Tooth Fractures" OR "Tooth Fracture" OR "Horizontal root fracture" OR "Transverse Root Fracture" OR "root fracture" OR "dental trauma" OR "dental injuries" OR "dental injury" OR "Dentoalveolar trauma" OR "intraalveolar root fracture" OR "intra alveolar root fracture" ) AND TITLE-ABS-KEY ( "splints" OR "splint" OR "Static Splints" OR "retention" OR "splinting" OR "Flexible splinting" OR "types of splints" OR "rigid splinting" OR "rigid splint" OR "flexible splint" OR "semi-rigid splinting" OR "tooth stabilization" OR "tooth splinting" OR "splinted" ) |
| **Web of Science** | 1. **TS=(“tooth injuries” OR “Teeth Injury” OR “tooth injury” OR “teeth injuries” OR "Tooth Fractures" OR “Tooth Fracture” OR "Horizontal root fracture" OR "Transverse Root Fracture" OR "root fracture" OR “dental trauma” OR “dental injuries” OR “dental injury” OR “Dentoalveolar trauma” OR “intraalveolar root fracture” OR “intra alveolar root fracture”)** 2. **TS=(“splints” OR “splint” OR “Static Splints” OR “retention” OR “splinting” OR “Flexible splinting” OR “types of splints” OR “rigid splinting” OR “rigid splint” OR “flexible splint” OR “semi-rigid splinting” OR “tooth stabilization” OR “tooth splinting” OR “splinted”)** 3. **#1 AND #2** |
| **LIVIVO** | **TI=(“tooth injuries” OR “Teeth Injury” OR “tooth injury” OR “teeth injuries” OR "Tooth Fractures" OR “Tooth Fracture” OR "Horizontal root fracture" OR "Transverse Root Fracture" OR "root fracture" OR “dental trauma” OR “dental injuries” OR “dental injury” OR “Dentoalveolar trauma”** OR “intraalveolar root fracture” OR “intra alveolar root fracture”**) AND TI=(“splints” OR “splint” OR “Static Splints” OR “retention” OR “splinting” OR “Flexible splinting” OR “types of splints” OR “rigid splinting” OR “rigid splint” OR “flexible splint” OR “semi-rigid splinting” OR “tooth stabilization” OR “tooth splinting” OR “splinted”)** |
| **Embase** | **('tooth injuries':ti,ab,kw OR 'teeth injury':ti,ab,kw OR 'tooth injury':ti,ab,kw OR 'teeth injuries':ti,ab,kw OR 'tooth fractures':ti,ab,kw OR 'tooth fracture':ti,ab,kw OR 'horizontal root fracture':ti,ab,kw OR 'transverse root fracture':ti,ab,kw OR 'root fracture':ti,ab,kw OR 'dental trauma':ti,ab,kw OR 'dental injuries':ti,ab,kw OR 'dental injury':ti,ab,kw OR 'dentoalveolar trauma':ti,ab,kw OR 'intraalveolar root fracture':ti,ab,kw OR 'intra alveolar root fracture':ti,ab,kw) AND ('splints':ti,ab,kw OR 'splint':ti,ab,kw OR 'static splints':ti,ab,kw OR 'retention':ti,ab,kw OR 'splinting':ti,ab,kw OR 'flexible splinting':ti,ab,kw OR 'types of splints':ti,ab,kw OR 'rigid splinting':ti,ab,kw OR 'rigid splint':ti,ab,kw OR 'flexible splint':ti,ab,kw OR 'semi-rigid splinting':ti,ab,kw OR 'tooth stabilization':ti,ab,kw OR 'tooth splinting':ti,ab,kw OR 'splinted':ti,ab,kw)** |
| **Cochrane Library** | (“tooth injuries” OR “Teeth Injury” OR “tooth injury” OR “teeth injuries” OR “Tooth Fractures” OR “Tooth Fracture” OR “Horizontal root fracture” OR “Transverse Root Fracture” OR “root fracture” OR “dental trauma” OR “dental injuries” OR “dental injury” OR “Dentoalveolar trauma” OR “intraalveolar root fracture” OR “intra alveolar root fracture” AND “splints” OR “splint” OR “Static Splints” OR “retention” OR “splinting” OR “Flexible splinting” OR “types of splints” OR “rigid splinting” OR “rigid splint” OR “flexible splint” OR “semi-rigid splinting” OR “tooth stabilization” OR “tooth splinting” OR “splinted”) |
| **Google Scholar** | (“tooth injuries” OR “Tooth Fractures”) AND (“Splints”) |
| **Open Grey** | (“tooth injuries” OR “Tooth Fractures”) AND (“Splints”) |
| **ProQuest** | (“tooth injuries” OR “Teeth Injury” OR “tooth injury” OR “teeth injuries” OR “Tooth Fractures” OR “Tooth Fracture” OR “Horizontal root fracture” OR “Transverse Root Fracture” OR “root fracture” OR “dental trauma” OR “dental injuries” OR “dental injury” OR “Dentoalveolar trauma” OR “intraalveolar root fracture” OR “intra alveolar root fracture” AND “splints” OR “splint” OR “Static Splints” OR “retention” OR “splinting” OR “Flexible splinting” OR “types of splints” OR “rigid splinting” OR “rigid splint” OR “flexible splint” OR “semi-rigid splinting” OR “tooth stabilization” OR “tooth splinting” OR “splinted”) |
