# Supplementary material for: Splinting in horizontal root fractures: A Bayesian network meta-analysis
Source: PLoS One. 2025 Jun 26;20(6):e0326979. doi: 10.1371/journal.pone.0326979 (PMC12200830; doi:10.1371/journal.pone.0326979)
Supplement: S2 Appendix — (DOCX) [file pone.0326979.s002.docx]

**Appendix 2 -** Studies excluded after being fully read with the reasons for exclusion (n = 21).

| **Author, Year** | **Reason for exclusion** |
| --- | --- |
| Wölner-Hanssen and von Arx, 2010 [1]  Verdugo-Avello, et al., 2014[2]  Qin, M., Ge, L., and Bai, R., 2002[3]  Molina, et al., 2008 [4]  Kirzioglu, et al., 2008 [5]  Fernández-Ferro, et al., 2020 [6]  Feely L, Mackie IC, Macfarlane T., 2003 [7]  Kim GT, et al., 2012 [8]  McIntosh, et al., 2009 [9]  Oulis, C. J., and Berdouses, E. D., 1996 [10]  Welbury, et al., 2002 [11]  Yassen GH, Chin JR, Al-Rawi BA, et al., 2013 [12]  Yates JA., 1992 [13]  Zaitoun, et al., 2010 [14]  Tan, et al., 2012 [15]  Bechtold H, Bull HG, Schubert F., 1987 [16]  Mackie IC, Blinkhorn AS., 1996 [17]  Cho, et al., 2018 [18]  Caliskan, M. K., and Pehlivan, Y., 1996 [19]  Miles, R. W., 2007 [20]  Cvek, M., Mejare, I., and Andreasen, J. O., 2004 [21] | 4  4  3  4  3  3  4  6  1  4  3  2  3  2  5  4  5  3  3  4  4 |

**Subtitle**

1) Studies in vitro (n = 1);

2) Etiological study (n = 2);

3) Studies that did not compared patients with different splinting materials (n = 7);

4) Studies in which none of the variables of interest have been evaluated (n = 8);

5) Reviews, letters, conference abstracts, personal opinions, case reports and case series (n = 2).

6) Studies that only included deciduous tooth ( n= 1).

**REFERENCES**

1. Wölner-Hanssen AB, von Arx T. Permanent teeth with horizontal root fractures after dental trauma: A retrospective study. Schweiz Monatsschr Zahnmed. 2010;120(3):200-212.
2. Verdugo-Avello FJ, González E, Pedemonte C, Vargas I. Fracturas radiculares en pacientes adultos: Propuesta de tratamiento actual. Rev Esp Cir Oral Maxilofac. 2014;36(2):54–58. <https://doi.org/10.1016/j.maxilo.2012.11.008>
3. Qin M, Ge L, Bai R. Use of a removable splint in the treatment of subluxated, luxated and root fractured anterior permanent teeth in children. Dent Traumatol. 2002;18(2):81–85. <https://doi.org/10.1034/j.1600-9657.2002.180207.x>
4. Molina JR, Vann WF, McIntyre JD, Trope M, Lee JY. Root fractures in children and adolescents: Diagnostic considerations. Dent Traumatol. 2008;24(5):503–509. <https://doi.org/10.1111/j.1600-9657.2008.00652.x>
5. Kirzioglu Z, Koseler Sentut T, Karayilmaz H, Ozay Erturk S. Case series: A clinical study of 27 cases of dentoalveolar root fractures in children and adolescents. Eur Arch Paediatr Dent. 2008;9(2):98–101. <https://doi.org/10.1007/BF03262618>
6. Fernández-Ferro M, Fernández-Sanromán J, Costas-López A, López-Betancourt A, Casañas-Villalba N, López-Fernández P. Complex dentoalveolar fractures: Main clinical variables description and analysis. J Craniofac Surg. 2020;31(8):e761–e765. <https://doi.org/10.1097/SCS.0000000000006711>
7. Feely L, Mackie IC, Macfarlane T. An investigation of root-fractured permanent incisor teeth in children. Dent Traumatol. 2003;19(1):52–54.
8. Kim GT, Sohn M, Ahn HJ, Lee DW, Choi SC. Intra-alveolar root fracture in primary teeth. Pediatr Dent. 2012;34(7):e215-e218.
9. McIntosh MS, Konzelmann J, Smith J, Kalynych CJ, Wears RL, Schneider H, et al. Stabilization and treatment of dental avulsions and fractures by emergency physicians using just-in-time training. Ann Emerg Med. 2009;54(4):585–592. https://doi.org/10.1016/j.annemergmed.2009.06.510
10. Oulis CJ, Berdouses ED. Dental injuries of permanent teeth treated in private practice in Athens. Endod Dent Traumatol. 1996;12(2):60–65. <https://doi.org/10.1111/j.1600-9657.1996.tb00098.x>
11. Welbury R, Kinirons MJ, Day P, Humphreys K, Gregg TA. Outcomes for root-fractured permanent incisors: A retrospective study. Pediatr Dent. 2002;24(2):98–102.
12. Yassen GH, Chin JR, Al-Rawi BAO, Mohammedsharif AG, Alsoufy SS, Hassan LA, et al. Traumatic injuries of permanent teeth among 6- to 12-year-old Iraqi children: A 4-year retrospective study.
13. Yates JA. Root fractures in permanent teeth: A clinical review. Int Endod J. 1992;25(3):150-157. https://doi.org/10.1111/j.1365-2591.1992.tb00778.x
14. Zaitoun H, North S, Lee S, Albadri S, McDonnell ST, Rodd HD. Initial management of paediatric dento-alveolar trauma in the permanent dentition: A multi-centre evaluation.
15. Voreaux P, Amans JM. [Reduction and retention in dental and alveolar fractures]. Rev Odontostomatol (Paris). 1987;6(5):391-394.
16. Bechtold H, Bull HG, Schubert F. Ergebnisse der transdentalen Stabilisierung gelockerter und wurzelfrakturierter Zähne [Results of transdental stabilization of loosened teeth and fractured tooth roots]. Dtsch Zahnarztl Z. 1987;42(3):295-298.
17. Mackie IC, Blinkhorn AS. Dental trauma: 3 splinting, displacement injuries and root fracture of immature permanent incisors. Dent Update. 1996;23(4):140-142.
18. Cho WC, Nam OH, Kim MS, Lee HS, Choi SC. A retrospective study of traumatic dental injuries in primary dentition: Treatment outcomes of splinting. Acta Odontol Scand. 2018;76(4):253-256. https://doi.org/10.1080/00016357.2017.1414956
19. Caliskan MK, Pehlivan Y. Prognosis of root-fractured permanent incisors. Dent Traumatol. 1996;12(3):129–136. https://doi.org/10.1111/j.1600-9657.1996.tb00111.x
20. Miles RW. Fallacious reasoning and complexity as root causes of clinical inertia. J Am Med Dir Assoc. 2007;8(6):349–354. https://doi.org/10.1016/j.jamda.2007.05.003
21. Cvek M, Mejare I, Andreasen JO. Conservative endodontic treatment of teeth fractured in the middle or apical part of the root. Dent Traumatol. 2004;20(5):261–269. <https://doi.org/10.1111/j.1600-9657>
